# Stability and Thrombogenicity Analysis of Collagen/Carbon Nanotube Nanocomposite Coatings Using a Reversible Microfluidic Device

**DOI:** 10.3390/membranes13040403

**Published:** 2023-04-01

**Authors:** Kristina D. Popovich, Sergey A. Vagner, Denis T. Murashko, Galina N. Ten, Dmitry I. Ryabkin, Mikhail S. Savelyev, Evgeny P. Kitsyuk, Ekaterina A. Gerasimenko, Polina Edelbekova, Anton N. Konovalov, Dmitry V. Telyshev, Sergey V. Selishchev, Alexander Yu. Gerasimenko

**Affiliations:** 1 Institute for Bionic Technologies and Engineering, I.M. Sechenov First Moscow State Medical University, Bolshaya Pirogovskaya Street 2-4, 119435 Moscow, Russia; 2 Institute of Biomedical Systems, National Research University of Electronic Technology, Shokin Square 1, Zelenograd, 124498 Moscow, Russia; 3 Department of Physics, Saratov State University, Astrakhanskaya Street 83, 410012 Saratov, Russia; 4 Scientific-Manufacturing Complex “Technological Centre”, Shokin Square 1, bld. 7 off. 7237, 124498 Moscow, Russia; 5 Orthopedic Department, State Autonomous Institution of Health of the City of Moscow, Dental Clinic No.35, Building 1638, Zelenograd, 124365 Moscow, Russia; 6 Insitute of Nanotechnology of Microelectronics of the Russian Academy of Sciences, 32a Leninsky Av., 119991 Moscow, Russia; 7 Burdenko Neurosurgical Center, 125047 Moscow, Russia

**Keywords:** nanocomposite, coating, microfluidic device, carbon nanotubes, collagen, thrombogenicity, hemocompatible membranes, blood, protein, bovine serum albumin, glutaraldehyde, adhesion

## Abstract

Currently, the development of stable and antithrombogenic coatings for cardiovascular implants is socially important. This is especially important for coatings exposed to high shear stress from flowing blood, such as those on ventricular assist devices. A method of layer-by-layer formation of nanocomposite coatings based on multi-walled carbon nanotubes (MWCNT) in a collagen matrix is proposed. A reversible microfluidic device with a wide range of flow shear stresses has been developed for hemodynamic experiments. The dependence of the resistance on the presence of a cross-linking agent for collagen chains in the composition of the coating was demonstrated. Optical profilometry determined that collagen/c-MWCNT and collagen/c-MWCNT/glutaraldehyde coatings obtained sufficiently high resistance to high shear stress flow. However, the collagen/c-MWCNT/glutaraldehyde coating was almost twice as resistant to a phosphate-buffered solution flow. A reversible microfluidic device made it possible to assess the level of thrombogenicity of the coatings by the level of blood albumin protein adhesion to the coatings. Raman spectroscopy demonstrated that the adhesion of albumin to collagen/c-MWCNT and collagen/c-MWCNT/glutaraldehyde coatings is 1.7 and 1.4 times lower than the adhesion of protein to a titanium surface, widely used for ventricular assist devices. Scanning electron microscopy and energy dispersive spectroscopy determined that blood protein was least detected on the collagen/c-MWCNT coating, which contained no cross-linking agent, including in comparison with the titanium surface. Thus, a reversible microfluidic device is suitable for preliminary testing of the resistance and thrombogenicity of various coatings and membranes, and nanocomposite coatings based on collagen and c-MWCNT are suitable candidates for the development of cardiovascular devices.

## 1. Introduction

According to the World Health Organization, approximately 17.9 million people around the world die from cardiovascular disease every year [1]. One of the most critical pathologies in this disease spectrum is heart failure. Heart failure is characterized by impaired myocardial function and the consequent inability of the heart to provide an adequate blood supply to the body due to the impaired pumping function of the heart [2]. Currently, the main methods of treatment of terminal heart failure are transplantation of a donor heart and implantation of a device that partially or completely replaces its function [3]. As there is an acute shortage of donor hearts in the world, a lot of attention is being paid to the second alternative, namely the creation of ventricular assist devices (VAD). One of these systems, the Sputnik-VAD, has been developed at the National Research University of Electronic Technology [4]. This device has been successfully used in medical practice and implanted in more than 50 patients. However, despite the increased survival rate and improved quality of life for patients, VAD implantation is associated with complications such as thrombosis and high hemolysis rates in some cases. To prevent thrombosis, the patients are usually given the common anticoagulant heparin. The anti-coagulation factor of heparin is determined by the presence of a pentasaccharide sequence (bioactive center) in the molecular structure [5]. This sequence neutralizes the effect of thrombin by binding it to antithrombin. However, the systematic treatment with anticoagulants is associated with the risk of thrombocytopenia and is generally ineffective if the patient has a congenital deficiency of antithrombin in the blood [6]. The World Health Organization reports that there is one person in every 5000 with an inherited deficiency of this protein.

One of the possible solutions to the described problems is to increase the hemocompatibility of the implanted device in contact with the blood. For this purpose, coatings capable of inhibiting thrombosis and consequently reducing the level of blood hemolysis can be used [7,8]. Coatings can also have an antibacterial effect to minimize the progression of infectious complications [9,10]. Coating methods include thermal spraying [11], magnetron sputtering [12], sol-gel method [13] and pulsed laser deposition [14]. Every one of these technologies has advantages and disadvantages [15]. Thermal spraying can achieve thicknesses from 30 to 200 μm at high speed and low cost. However, the disadvantage of this technology is the high temperatures, which can lead to decomposition of the coating or substrate. The magnetron sputtering method produces uniform coatings from 0.5 to 3 microns with a high degree of adhesion [16]. This technology is difficult to implement for coating parts with complex geometry, therefore it is characterized by a rather high cost. The sol-gel method can achieve layer thicknesses of <1 µm with a high degree of adhesion, but the process requires a careful approach to pressure and temperature control, and the use of expensive precursors [17,18]. Using the pulsed laser deposition method, it is possible to obtain a dense and porous coating with crystalline and amorphous phases of thickness from 0.05 to 5 µm [19].

More recently, carbon nanomaterials have been used in biomedical materials and membrane materials [20,21]. One of the most widespread carbon nanomaterials are carbon nanotubes (CNT). They have been widely and successfully used to create biocompatible functional coatings and three-dimensional implants with controllable surfaces and internal structures as well as mechanical, electrophysical and optical properties [22,23]. This is achieved by their small size, large specific surface area, and high stability CNT [24]. For homogeneous distribution in liquid solvents and solid matrices, CNT can be functionalized with specific chemical groups [25]. Depending on the medical application, different types of functional groups for nanotubes can be used [26]. Carboxyl, hydroxyl, and carbonyl groups result in water solubility and a good level of biocompatibility of nanotube-based materials [27]. To create biocompatible nanocomposites, CNT are functionalized with biopolymers, such as proteins [28]. Additionally, the enveloping of nanotubes in the polymer matrix allows us to reduce the effect of thrombogenicity [29]. The addition of CNT improves the hematological properties of materials for cardiovascular engineering [30,31]. Considering these facts, CNT are used as fillers in the collagen biopolymer matrix and membranes to create coatings providing a low level of blood hemolysis [32].

Collagen biopolymers have demonstrated great potential for use in various biomedical applications due to their high biocompatibility and low immunogenicity [33]. Collagen is actively used in cardiovascular regenerative medicine because it can be modified with drug molecules to achieve a gene therapy effect [34]. A complex of collagen and carbon nanotubes is used to form tissue-engineered materials to create cardiac patches for the myocardium [35]. Collagen-based coatings exhibit high hemocompatibility and anticoagulant effects with a low biodegradation rate (high stability) [36].

A variety of laboratory methods, usually performed under static or low shear conditions, are used as tools to evaluate the hemocompatibility of coatings [37]. This approach cannot provide reliable results because the mechanical effect of blood flow significantly affects the stability of the coating and the degree of thrombosis. This problem can be investigated and solved using a microfluidic device. By pumping a fluid through a microchannel of specified dimensions at different flow velocities, the proposed coatings can be studied in interaction with the flow. Such systems are used to create artificial vascular systems that mimic the geometry of natural vessels [38,39].

Therefore, the present work (i) proposed the method of forming nanocomposite coatings based on collagen and multi-walled carbon nanotubes that provide reduced thrombogenicity, (ii) proposed a microfluidic device to evaluate the effect of fluid flow on the developed coatings, and (iii) obtained the results of experimental studies on the resistance and anticoagulant properties of the coatings at high shear flow stresses.

## 2. Materials and Methods

### 2.1. Materials

The coating dispersion was prepared based on the bovine collagen type II (MacMedi LLC, Russia) and carbon nanotubes (Taunit, Tambov, Russia). The nanotubes were functionalized with carboxyl groups (COOH). The average diameter of the carboxylated multi-walled carbon nanotubes (c-MWCNT) was 10–30 nm, length ~1.8–22 μm, and the specific surface area was 270 m^2^/g. The degree of purity of c-MWCNT was 98%. Glutaric aldehyde (Sigma Aldrich, Missouri, MO, USA) was used to crosslink the polymer chains of the finished coatings. Phosphate-buffered solution (Sigma Aldrich, Missouri, MO, USA) and bovine serum albumin (BSA) solution (Biocloth BmbH, Munich, Germany) were used as a pumped liquid for experiments on the stability and anticoagulability of the coatings, respectively.

### 2.2. Preparation of Dispersions and Coatings

To form the coating, it was first necessary to prepare an aqueous dispersion of collagen and c-MWCNT. The dispersion was prepared in several steps. First, an aqueous suspension of collagen was prepared at a concentration of 1 wt.% in a weak solution of acetic acid (6%). Homogeneity of the solution was achieved by successive use of an ultrasonic bath (40 W) and a magnetic stirrer for 2 and 1 h, respectively. In the second step, an aqueous dispersion of c-MWCNT with a concentration of 0.01 wt.% was prepared. A submersible ultrasonic disperser (Qsonica Sonicators, Connecticut, CT, USA) was used for 40 min (20 W) to obtain a homogeneous dispersion of nanotubes. The collagen suspension was mixed with the c-MWCNT dispersion and subjected to a magnetic stirrer and an ultrasonic bath for 3 and 2 h, respectively. The result was the dispersion for the formation of the nanocomposite coating.

Next, 4 *×* 1.5 cm slide glass fragments were used as substrates for the nanocomposite coating. A 200 nm thick titanium layer was deposited on the glass substrates by magnetron sputtering. The sputtering of the titanium layer is a result of the fact that the developed coating is to be applied to the parts of the VAD, which are, as a rule, made of titanium. These VAD parts are in direct contact with blood. Before sputtering, all samples were ion blasted for 3 min to obtain high adhesion between the substrates and the titanium layer [40]. Magnetron sputtering was performed for 24 min at 1000 W and an argon pressure of 3 × 10^−^^3^ Tor. Subsequently, glass substrates with a titanium layer were plasma treated to increase the adhesion of the titanium layer with the nanocomposite coating [41]. Plasma treatment (100 cm^3^ of oxygen and 100 cm^3^ of argon) was performed for 1 min at 100 W at 280 °C. Thus, the substrates for nanocomposite coating were prepared.

**Figure 1:**
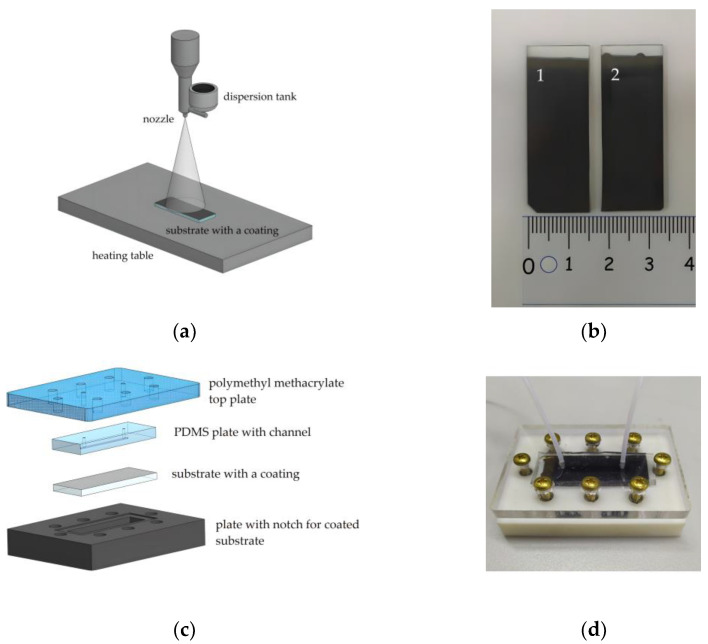
The scheme of aerosol layer-by-layer deposition (**a**); substrates with applied coatings based on collagen/c-MWCNT (1) and collagen/c-MWCNT/glutaraldehyde (2) (**b**); scheme (**c**) and photo (**d**) of the fabricated microfluidic chip.

The schematic representation of the collagen and c-MWCNT dispersion spraying unit is shown in Figure 1a. Dispersion was sprayed through a nozzle with an output diameter of 300 μm in the direction of the heating table. The nozzle was attached to the displacement module for dispersion spraying along the substrate. The automation of the multilayer coating spraying process with the specified characteristics of the nozzle movement (time, delay, and trajectory) was implemented using the Arduino hardware and software platform. Each coating sample was formed by applying 100 layers. A heating stage was used to evaporate the excess water at 70 ℃.

To crosslink the polymer chains, some of the coating samples were immersed in a solution of glutaric aldehyde (1%) for 30 min. Figure 1b shows the finished samples of collagen- and c-MWCNT-based coatings. In some cases, the use of glutaric aldehyde has disadvantages such as a tendency to calcification and thrombogenicity [42]. However, the usage of glutaric aldehyde is still clinically acceptable for coatings and membranes [43]. In this work, a study of the effect of glutaric aldehyde on the stability and thrombogenicity of collagen coatings was performed.

### 2.3. Microfluidic Device

The stability and anticoagulant properties of the nanocomposite coating in contact with the liquid flow were studied using a microfluidic device. The main element of the device was the microfluidic chip. The microfluidic chip required easy disassembly and reassembly to successfully study the interaction of the coating with the fluid flow, ensuring repeatability of the experiment. For this reason, the most versatile construction, shown in Figure 1, was chosen. The construction included four basic elements. Sealing of the microfluidic chip was achieved by screwing the outer elements (Figure 1c). The top element was made of polymethylmethacrylate. This element includes holes for tight connection of the fluid tubes and screws for fixing the structure. The lower element is made of polymer resin by 3D-printing. This element is necessary to fix the coated substrate with a special notch.

The inner element of the microfluidic chip with a channel was made of polydimethylsiloxane (PDMS) (SYLGARD 184, Dow Corning, Michigan, MI, USA). PDMS is known to be used for in vitro experiments, including experiments with biological fluids [44]. To form an element with a PDMS channel, a photopolymer mold was made for 3D printing casting (Wanhao E7+, Jinhua, China). The printed mold was further irradiated with ultraviolet (UV) light for the final polymerization. The printed mold contained an elevation to form a channel in the PDMS with height h = 250 μm, width ω = 1 mm, and length l = 25 mm. Then, the liquid PDMS was mixed with the crosslinking agent in a 1:10 ratio, thoroughly mixed, and poured into the printed mold. The filled mold was degassed in a vacuum chamber for 30 min and annealed in an oven at 70 °C for 4 h. After that, the PDMS had a stable dense-elastic state. The layer thickness of the PDMS was 4 mm, and the area coincided with the dimensions of the glass substrates.

Therefore, the microfluidic chip provided contact between the fluid flowing through the channel and the coating on the substrate. Figure 1d shows a photograph of the microfluidic chip assembly with the fluid flow inlet and outlet tubes connected. The fluid flow was controlled using an OB1 MK3+ pressure controller (ElveFlow, Paris, France). The MUX Recirculation 6/2 valve controller (ElveFlow, Paris, France) was used to organize the cyclic experiments.

### 2.4. Numerical Simulation Methodology

**Figure 2:**
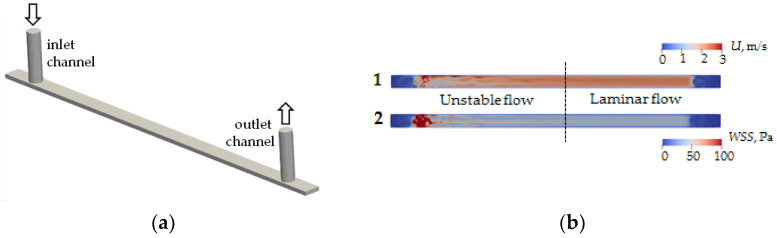
CAD model of the computing area of microfluidic chip channel (**a**) and modeling of velocity field distribution (1) and wall shear stress (2) of flow in a microfluidic chip channel (**b**).

Numerical simulations were performed to determine the type and structure of the flow inside the channel. Fluid flows into and out of the microchannel through microtubes with a diameter of 760 μm (Figure 2a). A CAD model was built using FreeCAD^®^ package (https://www.freecad.org (accessed on 31 March 2023)), and numerical calculation of the flow was performed using the finite volume method implemented in OpenFOAM^®^ version 9 (The OpenFOAM Foundation Ltd., London, UK). A hexagonal stack with the number of cells 793,952 was used in the calculations. Test calculations showed that further entrainment of the number of cells does not lead to qualitative or quantitative changes of velocity fields inside the channel and shear stresses on its walls. The calculation was performed for a fluid flow rate of 21 mL/min. The viscosity and density of the flowing liquid are 1 cSt and 1000 Pa/s, respectively.

### 2.5. Investigation of the Resistance of Coatings to the Flow

The main purpose of the experiment was to evaluate the effect of high shear stress on the surface of the nanocomposite coating. In this case, the shear stress was achieved by establishing the flow velocity. Experiments were performed at a flow rate of 21 mL/min and a pressure of 200 kPa.

VAD operate at high rpm, which results in high shear rates, above 100 s^−^^1^ [45]. This allows us to consider blood as a Newtonian fluid in the VAD, with a constant viscosity [46]. Therefore, a phosphate buffer aqueous solution with viscosity 1 cSt and density 1000 Pa/s was used as a model fluid. For this experiment, the important parameter of the model liquid was the acidity pH, in terms of its interaction with the surface. Therefore, the pH of the phosphate buffered aqueous solution used corresponded to the blood pH of 7.4.

Since we were interested in the effect of shear stress on the surface under investigation, it was decided to use a Newtonian fluid as a model fluid and vary the shear stress by increasing the fluid flow rate. The shear stresses on the walls of the rectangular cross-section microchannel can be estimated using the following equation [47]:

(1) $$WSS=\frac{6\mu Q}{wh^{2}}$$

where *µ* is the dynamic viscosity of the fluid (Pa·s), *Q* is the volumetric flow rate (m^3^/s), *w* is the channel width (m), and *h* is the channel height (m).

In accordance with Equation (1), the shear stresses on the flow side of the investigated coatings were 50 Pa. This value corresponds to the range of shear stress values in VAD, which can range from 0 to 150 Pa. It is known that at high values of shear stress (50 Pa), the process of platelet activation already occurs [48,49]. The experiments were carried out for 3.5 h. Every 30 min, the samples were removed from the microfluidic chip and examined on an S neox profilometer (SENSOFAR METROLOGY, Barcelona, Spain) with a CF60-2 20X objective (Nikon, Tokyo, Japan) to determine the resistance of the coating. In the experiments, each point was measured 5 times.

### 2.6. Investigation of the Thrombogenicity of Coatings

It is known that excessive appearance of blood albumin protein molecules adhered to the surface of vessels or implanted products is a precursor of thrombosis [50,51,52,53]. Therefore, the evaluation of BSA protein adhesion to the nanocomposite coating was used to determine the degree of thrombogenicity. BSA adhesion on the surface of the nanocomposite coating was determined using Raman spectroscopy and scanning electron microscopy.

The model aqueous solution contained blood protein BSA with a concentration of 40 g/L, which corresponds to the normal level of serum albumin in adult blood [54]. The solution viscosity was 0.88 mPa*s, which corresponded to the viscosity of adult blood [55]. The studied coatings were exposed to a flow of BSA solution in the microfluidic circuit for 30 min at a flow rate of 18 mL/min and a shear stress of 50 Pa.

Raman spectra, scanning electron microscopy (SEM) images, and energy-dispersive X-ray spectroscopy (EDX) for all coatings were measured in the original region before and after contact with the liquid flow. Five measurements were made for each area to obtain an average result.

Raman spectra were measured on a LabRAM HR Evolution instrument (Horiba, Essonne, France). An Ar laser with a wavelength of 514 nm and a power of ~0.125 mW was used as a source of excitation radiation. A diffraction grating of 1800 strokes/mm provided a spectral resolution of 0.5 cm^−^^1^. A precision motorized stage and a built-in BX41 microscope (Olympus, Shinjuku, Tokyo, Japan) were used to focus the laser beam on the studied area. The signal accumulation time was 15 *s* with averaging over 3 spectra to improve the signal-to-noise ratio.

A JEM-2100Plus (JEOL, Akishima, Tokyo, Japan) microscope was used to obtain SEM images at an electron column accelerating voltage of 5 kV and a current of 21 pA. EDX was performed using the Quantax XFlash 6 complex (Bruker, Massachusetts, MA, USA) with a modular system of backscattered electron diffraction in the vacuum chamber of the electron microscope.

## 3. Results and Discussion

### 3.1. Numerical Simulation

As a result of modeling, the velocity field in the cross-section at the half-height level of the microchannel h/2 (Figure 2b(1)) and wall shear stress on the lower wall of the microchannel (Figure 2b(2)) were calculated. It is found that the fluid flow entering perpendicular to the microchannel plane introduces significant fluctuations in velocity fields and shear stresses. In particular, the flow pattern inside the channel can be conditionally divided into two zones—an unstable flow zone and a laminar flow zone. The boundary between these zones corresponds approximately to the center of the microchannel. It follows from the results that to obtain the correct interaction results for given shear stresses on the flow side, the coverage analysis should be performed in the laminar flow zone.

### 3.2. Resistance of Coatings under the Influence of the Flow

**Figure 3:**
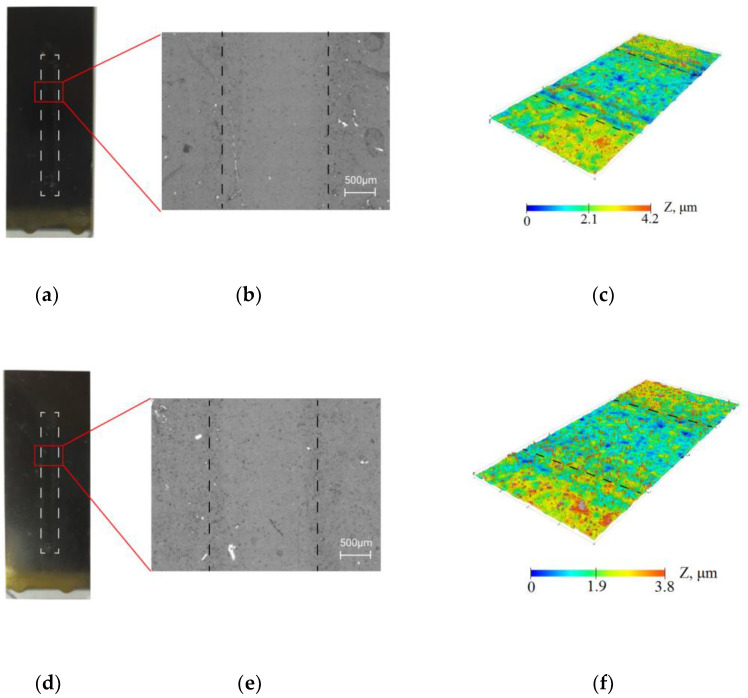
Resistance of collagen/c-MWCNT (**a**–**c**) and collagen/c-MWCNT/glutaraldehyde (**d**–**f**) coatings by optical microscopy (**b**,**e**) and profilometry (**c**,**f**) after contact with phosphate-buffered solution flow after 3.5 h.

The resistance of the collagen/c-MWCNT nanocomposite coatings (Figure 3a–c) and glutaraldehyde cross-linked collagen/c-MWCNT/glutaraldehyde (Figure 3d–f) was compared by exposure of the phosphate-buffered solution flow to the coating. The study of the resistance of the coatings was carried out using a non-destructive method—optical profilometry. Figure 3b,c,e,f show the surface analysis area of 1.77 × 3.65 mm. The analysis areas included a laminar flow contact zone and a coating area not exposed to the flow. The images and surface topography of the analysis area were measured after 3.5 h of flow exposure to the coating in the microfluidic device. A smaller elevation difference was obtained for the collagen/c-MWCNT/glutaraldehyde coating, indicating greater resistance of the glutaraldehyde cross-linked coating. The result indicates that glutaraldehyde stabilizes the coating.

**Figure 4:**
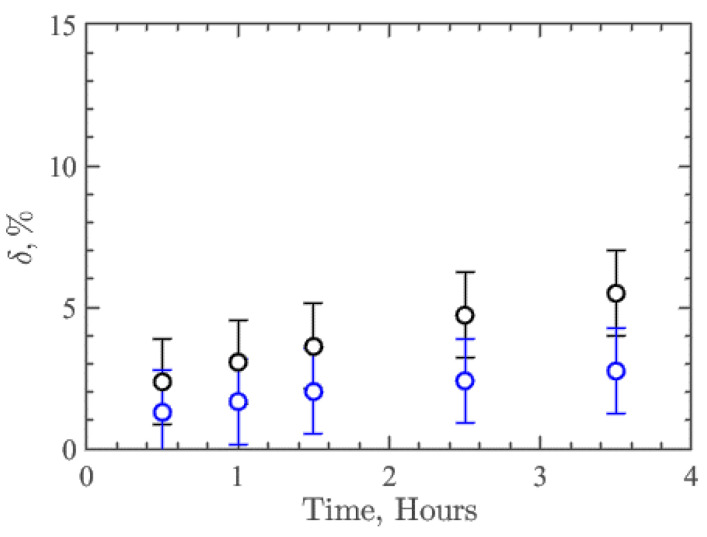
Dependence of the coatings collagen/c-MWCNT (black symbols) и collagen/c-MWCNT/glutaraldehyde (blue symbols) and degradation parameter δ on the time of contact with the flow in the microfluidic device.

The parameter δ was introduced to quantify the resistance/degradation rate over time:

(2) $$\delta =100\%*\left({\bar{z}}_{ch}-{\bar{z}}_{0}\right)/\bar{H}$$

where ${\bar{z}}_{ch}$—average height of the profile around contact with the flow, ${\bar{z}}_{0}$—the average height of the profile in the rest of the analysis area, which is not affected by the flow, and $\bar{H}$—the average thickness of the coatings, which was 10 µm. Thus, this parameter δ characterizes the relative resistance or degree of degradation of the nanocomposite coating. Figure 4 shows the results of δ dependence on time for collagen/c-MWCNT (black symbols) and collagen/c-MWCNT/glutaraldehyde (blue symbols) nanocomposite coatings. The resistance of collagen/c-MWCNT/glutaraldehyde coating (δ = 2.75%) is almost two times higher than that of collagen/c-MWCNT (δ = 5.5%) after 3.5 h of flux exposure. However, for both types of coatings, a rather high resistance to high shear stress flow was obtained.

### 3.3. Thrombogenicity

During blood flow in VAD, blood proteins first adhere to the surface of the implanted part. These proteins can be divided into two groups. The first group includes proteins that adhere to the surface first, and the second group includes proteins that attach to proteins from the first group. This phenomenon is called the Vroman effect [56]. In the study of surfaces after contact with blood, the presence of the highest concentration of such proteins as albumin, fibrinogen, immunoglobulins, vitronectin and apolipoproteins was determined in most cases. It was found that platelet adhesion depends on the adsorption to the surface of the listed proteins [57,58]. In this case, receptors on the surface of platelets interact with certain amino acid sequences in the adsorbed proteins. The shape of the adherent platelets changes greatly from disc-shaped to pseudopodial. Then, there is a release of platelet contents: platelet factor 4, adenosine diphosphate, serotonin, and platelet aggregates formation. The subsequent activation of coagulation factors, proteins found in platelets and plasma, leads to the formation of thrombin. Thrombin, in turn, awakens the fibrin protein from the inactive state of the fibrinogen protein. As a result, the formed fibrin network holds platelets and other blood cells in place, ensuring the formation of the thrombus. The highest concentration of all blood proteins is in albumin, which has a transport function in the body. The concentration of albumin is 35–53 g/L [59]. Therefore, the degree of adhesion of albumin to the coating surface or membranes can be considered an initial test for thrombogenicity.

In the experiments to study the degree of adhesion of albumin to the surface of nanocomposite coatings, an aqueous solution with a BSA concentration of ~64 g/L was used to provide the necessary viscosity. In this model solution, albumin was the only dissolved substance that tended to adhere to the surface.

The thrombogenicity of the coatings was investigated by comparing Raman spectra and SEM with energy dispersive spectroscopy of the coating before and after contact with the flow of the BSA solution. At the same time, the degree of adhesion of BSA to nanocomposite coatings was compared with the adhesion of BSA to titanium, from which VAD are fabricated.

**Figure 5:**
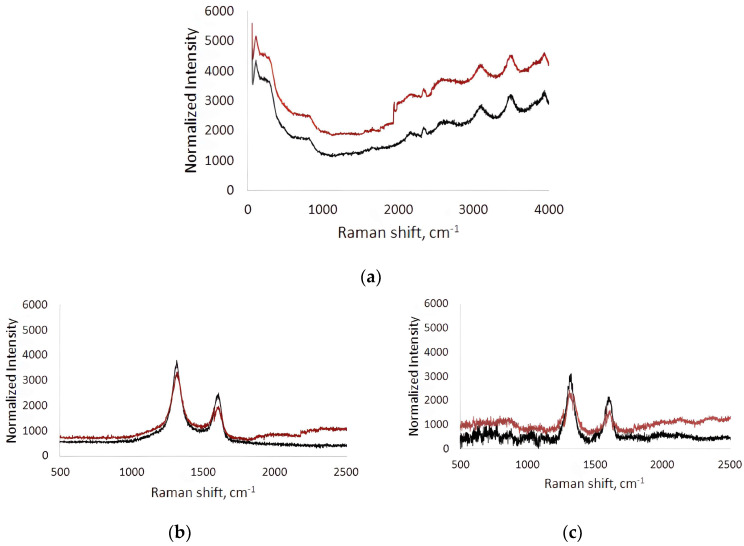
Raman spectra of titanium surface (**a**), collagen/c-MWCNT nanocomposite coatings (**b**) and collagen/c-MWCNT/glutaraldehyde (**c**) before (black line) and after (red line) contact with BSA solution flow.

Raman spectra were recorded before (black line) and after (red line) contact of the BSA solution flow in the microfluidic chip with the surface (Figure 5). The spectra were measured at five different points in the analysis area and averaged. The Raman spectra of the titanium surface after contact with the BSA solution flow compared to the original titanium surface were characterized by an increase in fluorescence over a wide frequency range (Figure 5a). This indicates the contribution of BSA molecules adhered to the titanium surface to the fluorescence response. On average, the increase in fluorescence was 18–36%.

In the Raman spectra of the collagen/c-MWCNT nanocomposite coatings (Figure 5b) and collagen/c-MWCNT/glutaraldehyde (Figure 5c), characteristic modes for multi-walled carbon nanotubes (D- and G-modes) were observed. D-mode arises due to the process of double resonant Raman light scattering [60]. D-mode is observed in the presence of defects in the graphene/graphite structure; it has an intensity that is proportional to the degree of disorder in the MWNT structure [61]. The G-mode corresponds to planar optical phonon modes. This mode is characteristic of all carbon materials with sp_2_-hybridization. In the case of bond breaking in the graphene structure forming the tube, atoms with sp_3_-hybridized electrons appear outside the plane of the nanotube layers. In this case, the atoms fluctuate with the frequency of the D-mode rather than the G-mode, therefore its intensity decreases in structures with a high level of defectiveness. The ratio of G- and D-mode bands intensity (I_G_/I_D_) is used in MWCNT defect analysis [62]. I_G_/I_D_ for collagen/c-MWCNT was 0.53, and for collagen/c-MWCNT/glutaraldehyde I_G_/I_D_ = 0.57. At the same time, the ratio of I_G_/I_D_ for the initial nanotube powder was 0.54. It can be concluded that no significant changes in the structure of the nanotubes were detected when the nanocomposite coating was made of them. Additionally, the defectiveness of the nanotubes in the coating was practically unchanged after interaction with the BSA flow. Thus, the magnitude of changes in the Raman spectrum of nanocomposite coatings after their contact with the flow of the BSA solution can be used as a characteristic of blood proteins’ adhesion to the coating.

Comparison of the Raman spectra of the collagen/c-MWCNT nanocomposite coatings and collagen/c-MWCNT/glutaraldehyde before and after contact with the BSA solution flow allowed the following conclusions: (i) the total fluorescence background increased, and (ii) the intensity of D- and G-mode peaks changed on the dependences for both coatings. Adhesion of BSA to the collagen/c-MWCNT coating up to 1000 cm^−1^ resulted in an increase in fluorescence to 13% and in the 1800–2500 cm^−1^ region to 18%. At the same time, the intensity of D- and G-mode peaks decreased by 11% and 13%, respectively. The decrease in the intensity of D- and G-mode peaks indicates the appearance of an albumin layer on the coating surface, which leads to the attenuation of the scattered signal from nanotubes. The amount of BSA on the collagen/c-MWCNT/glutaraldehyde nanocomposite coating was greater: up to 1000 cm^−1^ the increase in fluorescence reached 15%, and up to 24% in the 1800–2500 cm^−1^ region. The intensity of the D- and G-mode peaks from MWCNT in the coverage decreased by 14% and 20%, respectively. It was found that glutaric aldehyde in the composition of the nanocomposite coating provides slightly higher adhesion of BSA. At the same time, the highest level of adhesion was obtained for the titanium surface. From the comparison of the average fluorescence level, a preliminary conclusion can be made that BSA adhesion to collagen/c-MWCNT and collagen/c-MWCNT/glutaraldehyde coatings is 1.7 and 1.4 times less than to the titanium surface, i.e., such coatings are the least thrombogenic compared to the classic titanium surface.

**Figure 6:**
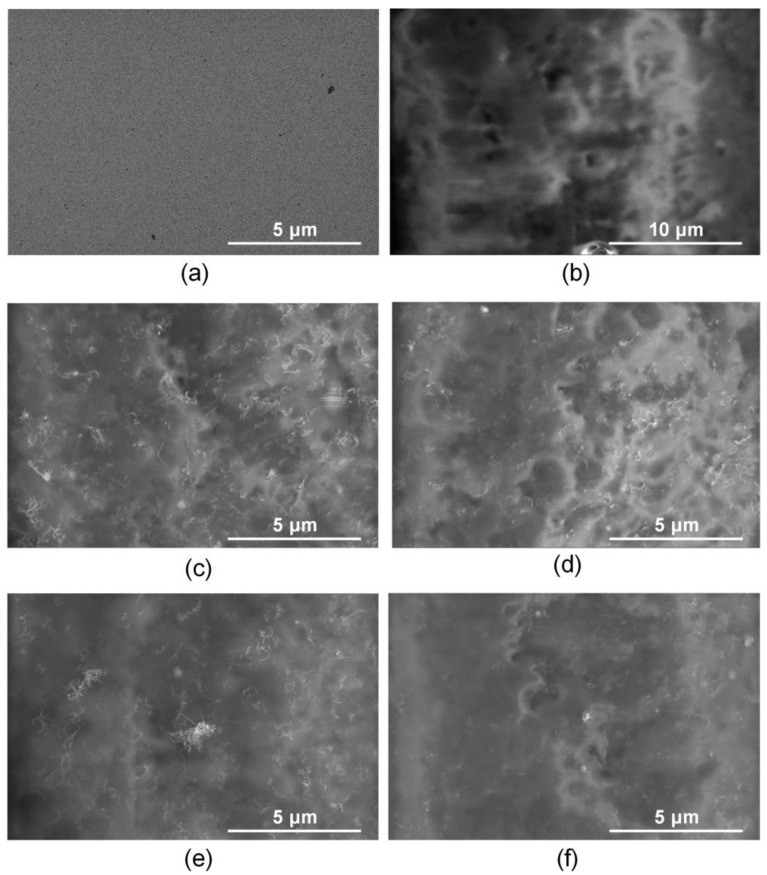
SEM images of titanium surface (**a**,**b**), nanocomposite coatings collagen/c-MWCNT (**c**,**d**) and collagen/c-MWCNT/glutaraldehyde (**e**,**f**) before (**a**,**c**,**e**) and after (**b**,**d**,**f**) contact with BSA solution flow.

Figure 6 shows SEM images for the titanium surface, the collagen/c-MWCNT and collagen/c-MWCNT/glutaraldehyde nanocomposite coatings before and after contact with the BSA solution.

The initial titanium surface is characterized by a uniform planar structure (Figure 6a). An irregular layer of blood protein is observed on the titanium surface after contact with the BSA solution (Figure 6b). Nanotube inclusions in the collagen matrix are observed on the surface of the collagen/s-MWCNT coating (Figure 6c). After contact with a flow of BSA solution, the surface of the coating was almost unchanged (Figure 6d). The nanotubes are still clearly visible on the surface. A slight change in the surface structure caused by the adhesion of a small amount of BSA is shown. The initial surface of the collagen/c-MWCNT/glutaraldehyde coating (Figure 6e) is similar to the collagen/c-MWCNT surface (Figure 6c). The cross-linking of the collagen chains with glutaraldehyde has almost no effect on the surface structure. However, after contact with BSA, the nanotubes are faintly visible in the SEM image (Figure 6f). This fact could be caused by the adhesion of the blood protein layer.

The BSA layer on the surface of the coatings and membranes can be quantified using the EDX method. This method identifies the chemical composition of set points for the same areas imaged by SEM. EDX was measured at several points for each sample and the average values were calculated. Table 1 shows the results of the changes in the chemical composition of the coatings before and after adsorption of blood protein to the surface.

Table 1 shows the change in the concentration of a number of chemical elements on the titanium surface and on the nanocomposite coatings. The amount of Ti after contact with BSA was practically halved and amounted to 12.44 wt.%. At the same time, elements such as C, Na, P, and S appeared, and the concentration of N and O increased by 5.3 and 1.5 times, respectively. The attenuation of the signal by titanium, the appearance of new elements, and an increase in the concentration of elements characteristic of BSA may indicate the appearance of a blood protein layer on the titanium surface.

The C signal is mainly provided by the contribution of nanotubes. The slight decrease in the carbon signal for both types of coatings is probably due to the appearance of albumin. The Ti and C signal was found to be almost halved in the case of collagen/c-MWCNT/glutaraldehyde coating compared to collagen/c-MWCNT. An increase in the concentration of the elements C, N, O, Na, P, and S was also obtained for both nanocomposite coatings. The signal from impurities included in the c-MWCNT composition was not detected by the EDX method. This is due to the high purity of the nanotubes and the low concentration of impurities in the composition of the coating volume (not more than 0.0002 wt.%).

It can be concluded that the highest BSA adhesion is obtained on the titanium surface. In second place is the collagen/c-MWCNT/glutaraldehyde coating with significantly lower albumin adhesion. Additionally, blood protein was least detected on the collagen/c-MWCNT coating.

## 4. Conclusions

During implantation, blood proteins adhere to titanium without nanocomposite coatings, which direct the subsequent activation of blood, which determines the next cascade of biological reactions and, consequently, the long-term success of using implantable devices. At the same time, the use of antithrombotic drugs increases the risk of bleeding and therefore this method is not complete. All this indicates the need to improve anticoagulant properties. This work proposes a method for the fabrication of coatings for ventricular assist devices or other implantable blood pumping devices. The coating has a nanocomposite structure of a collagen matrix with a distributed framework of carboxylated MWCNT. The coating or membranes were spread by spray deposition layer by layer. Glutaraldehyde as a crosslinking agent has the disadvantages of being prone to calcification and thrombogenicity, but its use is still clinically acceptable. In this work, it is shown that in the case of using crosslinking of the developed nanocomposite coating with glutaraldehyde, there is still a reduced thrombogenicity compared with titanium, which is a characteristic feature of such a coating. The results of comparing the resistance and thrombogenicity of collagen/c-MWCNT and collagen/c-MWCNT/glutaraldehyde crosslinked chain coatings are presented. To test coatings, a microfluidic device was developed that provides fluidic contact with easily replaceable coating samples, allowing us to conduct research in dynamic conditions. The reversible microfluidic device is suitable for preliminary testing and selection of coatings for further studies. Using the microfluidic device and optical profilometry, both types of coatings were found to have sufficiently high resistance to high shear flow. However, the collagen/c-MWCNT/glutaraldehyde coating (δ = 2.75%) was almost twice as stable as the collagen/c-MWCNT coating (δ = 5.5%) after 3.5 h of exposure to the phosphate-buffered solution flow. A reversible microfluidic device allowed the study of the interaction of blood protein albumin solution, and the adhesion on the surface of the coating indicates the degree of thrombogenicity. Using Raman spectroscopy, it was found that the adhesion of BSA to collagen/c-MWCNT and collagen/c-MWCNT/glutaraldehyde coatings is 1.7 and 1.4 times less than that to the titanium surface, which is widely used for ventricular assist devices. In addition, scanning electron microscopy and energy dispersive spectroscopy revealed that the greatest adhesion of blood protein was on the titanium surface compared to the nanocomposite coatings. At the same time, albumin was least detected on the collagen/s-MWCNT coating, which did not contain the collagen chain cross-linking agent—glutaric aldehyde.

Inspired by our results, we plan to investigate the compatibility of the manufactured composite coatings with blood, namely, to evaluate the degree of hemolysis upon contact of the coatings with blood in the developed microfluidic device. At this stage, the study already shows that nanocomposite coatings are potentially promising candidates for reducing the thrombogenicity of cardiovascular device surfaces and membranes.

## Figures and Tables

**Table 1:** Chemical composition of titanium surface samples and collagen/c-MWCNT and collagen/c-MWCNT/glutaraldehyde nanocomposite coatings before and after contact with BSA solution flow.

Chemical Elements	Titanium Surface (wt.%)			Coating Collagen/c-MWCNT (wt.%)			Coating Collagen/c-MWCNT/Glutaraldehyde (wt.%)		
	Before	After	Difference	Before	After	Difference	Before	After	Difference
Ti	23.96	12.44	−11.52	12.02	10.76	−1.26	11.74	9.35	−2.39
C	-	3.02	3.02	18.36	16.18	−2.18	16.53	13.18	−3.35
N	2.62	13.95	11.33	1.23	4.68	3.45	1.78	8.65	6.87
O	8.09	12.37	4.28	9.47	11.98	2.51	15.69	18.92	3.23
Na	-	0.41	0.41	0.11	0.26	0.15	0.09	0.37	0.28
P	-	0.07	0.07	-	0.05	0.05	-	1.12	1.12
S	-	1.16	1.16	0.16	1.17	1.17	0.12	1.39	1.27

## Data Availability

Not applicable.

## References

[1] Ghosh P., Azam S., Jonkman M., Karim A., Shamrat F.J.M., Ignatious E., Shultana S., Beeravolu A.R., De Boer F. (2021). Efficient Prediction of Cardiovascular Disease Using Machine Learning Algorithms with Relief and LASSO Feature Selection Techniques. IEEE Access.

[2] Crespo-Leiro M.G., Metra M., Lund L.H., Milicic D., Costanzo M.R., Filippatos G., Gustafsson F., Tsui S., Barge-Caballero E., Jonge N. (2018). Advanced heart failure: A position statement of the Heart Failure Association of the European Society of Cardiology. Eur. J. Heart Fail..

[3] Habal M.V., Garan A.R. (2017). Long-term Management of End-Stage Heart Failure. Best Pract. Res. Clin. Anaesthesiol..

[4] Telyshev D., Denisov M., Markov A., Fresiello L., Verbelen T., Selishchev S. (2019). Energetics of Blood Flow in Fontan Circulation under VAD Support. Artif. Organs.

[5] Braune S., Latour R.A., Reinthaler M., Landmesser U., Lendlein A. (2019). In Vitro Thrombogenicity Testing of Biomaterials. Adv. Healthc. Mater..

[6] Zang L., Zhu H., Wang K., Liu Y., Yu F., Zhao W. (2022). Not Just Anticoagulation—New and Old Applications of Heparin. Molecules.

[7] Liu X., Yuan L., Li D., Tang Z., Wang Y., Chen G., Chen H., Brash J.L. (2014). Blood Compatible Materials: State of the Art. J. Mater. Chem. B.

[8] Henkes H., Bhogal P., Pérez M.A., Lenz-Habijan T., Bannewitz C., Peters M., Sengstock C., Ganslandt O., Lylyk P., Monstadt H. (2019). Anti-thrombogenic Coatings for Devices in Neurointerventional Surgery: Case Report and Review of the Literature. Interv. Neuroradiol..

[9] Jin Y., Zhu Z., Liang L., Lan K., Zheng Q., Wang Y., Guo Y., Zhu K., Mehmood R., Wang B. (2020). A Facile Heparin/carboxymethyl Chitosan Coating Mediated by Polydopamine on Implants for Hemocompatibility and Antibacterial Properties. Appl. Surf. Sci..

[10] Rahvar M., Lakalayeh G.A., Nazeri N., Karimi R., Borzouei H., Ghanbari H. (2022). Micro/nanoscale Surface Engineering to Enhance Hemocompatibility and Reduce Bacterial Adhesion for Cardiovascular Implants. Mater. Chem. Phys..

[11] Shimazaki T., Miyamoto H., Ando Y., Noda I., Yonekura Y., Kawano S., Miyazaki M., Mawatari M., Hotokebuchi T. (2009). In Vivo Antibacterial and Silver-Releasing Properties of Novel Thermal Sprayed Silver-Containing Hydroxyapatite Coating. J. Biomed. Mater. Res. Part B Appl. Biomater..

[12] Akhavan O., Ghaderi E. (2009). Enhancement of Antibacterial Properties of Ag Nanorods by Electric Field. Sci. Technol. Adv. Mater..

[13] Akhavan O. (2009). Lasting Antibacterial Activities of Ag-TiO2/Ag/a-TiO2 Nanocomposite Thin Film Photocatalysts Under Solar Light Irradiation. J. Colloid Interface Sci..

[14] Pradhaban G., Kaliaraj G.S., Vishwakarma V. (2014). Antibacterial Effects of Silver–Zirconia Composite Coatings Using Pulsed Laser Deposition onto 316L SS for Bio Implants. Prog. Biomater..

[15] Gan J.A., Berndt C.C. (2015). Nanocomposite Coatings: Thermal Spray Processing, Microstructure and Performance. Int. Mater. Rev..

[16] Tan X.Q., Liu J.Y., Niu J.R., Liu J.Y., Tian J.Y. (2018). Recent Progress in Magnetron Sputtering Technology Used on Fabrics. J. Mater..

[17] Danks A.E., Hall S.R., Schnepp Z. (2016). The Evolution of ‘Sol–gel’ Chemistry as a Technique for Materials Synthesis. Mater. Horiz..

[18] Priyadarshini B., Priyadarshini B., Murugupandian R. (2019). Bioactive Coating as a Surface Modification Technique for Biocompatible Metallic Implants: A Review. J. Asian Ceram. Soc..

[19] Yang Z., Hao J. (2016). Progress in Pulsed Laser Deposited Two-dimensional Layered Materials for Device Applications. J. Mater. Chem. C.

[20] Jalilinejad N., Rabiee M., Baheiraei N., Ghahremanzadeh R., Salarian R., Rabiee N., Akhavan O., Zarrintaj P., Hejna A., Saeb M.R. (2023). Electrically Conductive Carbon-Based (Bio)-Nanomaterials for Cardiac Tissue Engineering. Bioeng. Transl. Med..

[21] Teow Y.H., Mohammad A.W., Ang W.L., Lee P.H. (2018). Development of Graphene Oxide (GO)/Multi-Walled Carbon Nanotubes (MWCNTs) Nanocomposite Conductive Membranes for Electrically Enhanced Fouling Mitigation. J. Membr. Sci..

[22] Gerasimenko A.Y., Kurilova U.E., Suetina I.A., Mezentseva M.V., Zubko A.V., Sekachev M., Glukhova O.E. (2021). Laser Technology for the Formation of Bioelectronic Nanocomposites Based on Single-Walled Carbon Nanotubes and Proteins with Different Structures, Electrical Conductivity and Biocompatibility. Appl. Sci..

[23] Demidenko N.A., Kuksin A.V., Molodykh V.V., Pyankov E.S., Ichkitidze L.P., Zaborova V., Tsymbal A.A., Tkachenko S.A., Shafaei H., Diachkova E. (2022). Flexible Strain-Sensitive Silicone-CNT Sensor for Human Motion Detection. Bioengineering.

[24] Simon J., Flahaut E., Golzio M. (2019). Overview of Carbon Nanotubes for Biomedical Applications. J. Mater..

[25] Kim S.W., Kim T., Kim Y.S., Choi S.H., Lim H.J., Kim Y.J., Choi S.Y. (2012). Surface Modifications for the Effective Dispersion of Carbon Nanotubes in Solvents and Polymers. Carbon.

[26] Negri V., Pacheco-Torres J., Calle D., López-Larrubia P. (2020). Carbon Nanotubes in Biomedicine. Topics in Current Chemistry Collections.

[27] Zhou L., Forman H., Yi G., Lunec G. (2017). Multi-Walled Carbon Nanotubes: A Cytotoxicity Study in Relation to Functionalization, Dose and Dispersion. Toxicol. Vitr..

[28] Gerasimenko A., Ten G.N., Ryabkin D., Shcherbakova N.E., Morozova E., Ichkitidze L.P. (2019). The Study of the Interaction Mechanism between Bovine Serum Albumin and Single-Walled Carbon Nanotubes Depending on Their Diameter and Concentration in Solid Nanocomposites by Vibrational Spectroscopy. Spectrochim. Acta—Part A Mol. Biomol. Spectrosc..

[29] Vakhrusheva T.V., Gusev A.A., Gusev S.A., Vlasova I.I. (2013). Albumin Reduces Thrombogenic Potential of Single-walled Carbon Nanotubes. Toxicol. Lett..

[30] Gerasimenko A.Y., Kitsyuk E., Kurilova U.E., Suetina I.A., Russu L., Mezentseva M.V., Markov A., Narovlyansky A.N., Kravchenko S., Selishchev S.V. (2022). Interfaces Based on Laser-Structured Arrays of Carbon Nanotubes with Albumin for Electrical Stimulation of Heart Cell Growth. Polimers.

[31] Gerasimenko A.Y., Kurilova U.E., Savelyev M.S., Murashko D., Glukhova O.E. (2021). Laser Fabrication of Composite Layers from Biopolymers with Branched 3D Networks of Single-walled Carbon Nanotubes for Cardiovascular Implants. Compos. Struct..

[32] Gerasimenko A.Y., Zhurbina N.N., Cherepanova N.G., Semak A.E., Zar V.V., Fedorova Y.O., Eganova E.M., Pavlov A., Telyshev D., Selishchev S.V. (2020). Frame Coating of Single-Walled Carbon Nanotubes in Collagen on PET Fibers for Artificial Joint Ligaments. Int. J. Mol. Sci..

[33] Primavera R., Razavi M., Kevadiya B.D., Wang J., Vykunta A., Mascolo D.D., Decuzzi P., Thakor A.S. (2021). Enhancing Islet Transplantation Using a Biocompatible Collagen-PDMS Bioscaffold Enriched with Dexamethasone-Microplates. Biofabrication.

[34] Zhang D., Wu X., Chen J., Lin K. (2018). The Development of Collagen Based Composite Scaffolds for Bone Regeneration. Bioact. Mater..

[35] Tashakori-Miyanroudi M., Rakhshan K., Ramez M., Asgarian S., Janzadeh A., Azizi A., Seifalian A., Ramezani F. (2020). Conductive Carbon Nanofibers Incorporated into Collagen Bio-scaffold Assists Myocardial Injury Repair. Int. J. Biol. Macromol..

[36] Cherng W.J., Pan Y.H., Wu T.C., Chou C.C., Yeh C.H., Ho J.J. (2019). Hemocompatibility and Adhesion of Heparin/Dopamine and Heparin/Collagen Self-assembly Multilayers Coated on a Titanium Substrate. Appl. Surf. Sci..

[37] Mohan C.C., Chennazhi K.P., Menon D. (2013). In Vitro Hemocompatibility and Vascular Endothelial Cell Functionality on Titania Nanostructures under Static and Dynamic Conditions for Improved Coronary Stenting Applications. Acta Biomater..

[38] Fenech M., Girod V., Claveria V., Meance S., Abkarian M., Charlot B. (2019). Microfluidic Blood Vasculature Replicas Using Backside Lithography. Lab Chip.

[39] Pinto E.A., Faustino V., Rodrigues R.O., Pinho D., Garcia V., Miranda J., Lima R.A. (2014). Rapid and Low-cost Nonlithographic Method to Fabricate Biomedical Microdevices For Blood Flow Analysis. Micromachines.

[40] Wei J., Zhu X.Y., Chen L.X., Liu J., Hei L., Li C., Zhang Y. (2015). Influence of Ion Source Pretreatment on WC Substrate Surface and Ta Buffer Coating. Cailiao Rechuli Xuebao/Trans. Mater. Heat Treat..

[41] Abdelaal A.F., Samad M.A., Adesina A.Y., Baig M.M.A. (2022). Effect of Plasma Treatment on the Tribological and Adhesion Performance of a Polymer Coating Deposited on Different Metallic Substrates. J. Coat. Technol. Res..

[42] Yang F., Xu L., Kuang D., Ge Y., Guo G., Wang Y. (2021). Polyzwitterion-crosslinked Hybrid Tissue with Antithrombogenicity, Endothelialization, Anticalcification Properties. Chem. Eng. J..

[43] Bigi A., Cojazzi G., Panzavolta S., Rubini K., Roveri N. (2001). Mechanical and Thermal Properties of Gelatin Films at Different Degrees of Glutaraldehyde Crosslinking. Biomaterials.

[44] Kim S., Ye S.-h., Adamo A., Orizondo R.A., Jo J., Cho S.K., Wagner W.R. (2020). A Biostable, Anti-fouling Zwitterionic Polyurethane-Urea Based on PDMS for Use in Blood-Contacting Medical Devices. J. Mater. Chem. B.

[45] Fraser K.H., Zhang T., Taskin M.E., Griffith B.P., Wu Z.J. (2012). A Quantitative Comparison of Mechanical Blood Damage Parameters in Rotary Ventricular Assist Devices: Shear Stress, Exposure Time and Hemolysis Index. J. Biomech. Eng..

[46] Papaioannou T.G., Stefanadis C. (2005). Vascular Wall Shear Stress: Basic Principles and Methods. Hellenic. J. Cardiol..

[47] Pisapia F., Balachandran W., Rasekh M. (2022). Organ-on-a-Chip: Design and Simulation of Various Microfluidic Channel Geometries for the Influence of Fluid Dynamic Parameters. Appl. Sci..

[48] Thamsen B., Blümel B., Schaller J., Paschereit C.O., Affeld K., Goubergrits G., Kertzscher U. (2015). Numerical Analysis of Blood Damage Potential of the HeartMate II and HeartWare HVAD Rotary Blood Pumps. Artif. Organs.

[49] Bui A.V., Manasseh R., Liffman K., Sutalo I.D. (2010). Development of Optimized Vascular Fractal Tree Models Using Level Set Distance Function. Med. Eng. Phys..

[50] Regan-Smith S., Fritzen R., Hierons S.J., Ajjan A.A., Blindauer C.A., Stewart A. (2022). Strategies for Therapeutic Amelioration of Aberrant Plasma Zn2+ Handling in Thrombotic Disease: Targeting Fatty Acid/Serum Albumin-Mediated Effects. Int. J. Mol. Sci..

[51] Liu K., Chen J., Zhang K., Wang S., Li X. (2019). A Diagnostic Prediction Model of Acute Symptomatic Portal Vein Thrombosis. Ann. Vasc. Surg..

[52] Milleret M., Buzzi S., Gehrig P., Ziogas A., Grossmannc J., Schilcher K., Zinkernagel A.S., Zucker A., Ehrbar M. (2015). Protein Adsorption Steers Blood Contact Activation on Engineered Cobalt Chromium Alloy Oxide Layers. Acta Biomater..

[53] Bulwan M., Wojcik K., Zapotoczny S., Nowakowska M. (2012). Chitosan-Based Ultrathin Films as Antifouling, Anticoagulant and Antibacterial Protective Coatings. Biomater. Sci..

[54] Rodriguez-Segade S., Rodriguez J., Mayan D., Camina F. (2005). Plasma Albumin Concentration Is a Predictor of HbA1c Among Type 2 Diabetic Patients, Independently of Fasting Plasma Glucose and Fructosamine. Diabetes Care.

[55] Shadiow J.A., Tarumi T., Dhindsa M., Hunter S.D. (2019). Comparison of Blood Viscosity and Hematocrit Levels between Yoga Practitioners and Sedentary Adults. Int. J. Exerc. Sci..

[56] Hirsh S.L., McKenzie D.R., Nosworthy N.J., Denman J.A., Sezerman O.U., Bilek M.M.M. (2013). The Vroman Effect: Competitive Protein Exchange with Dynamic Multilayer Protein Aggregates. Colloids Surf. B Biointerfaces.

[57] De Mel A., Cousins B.G., Seifalian A.M. (2012). Surface Modification of Biomaterials: A Quest for Blood Compatibility. Int. J. Biomater..

[58] Brash J.L., Horbett T.A., Latour R.A., Tengvall T. (2019). The Blood Compatibility Challenge. Part 2: Protein Adsorption Phenomena Governing Blood Reactivity. Acta Biomater..

[59] Nevídalová N., Michalcová L., Glatz Z. (2020). Applicability of Capillary Electrophoresis-Frontal Analysis for Displacement Studies: Effect of Several Drugs on l-tryptophan and Lidocaine Binding to Human Serum Albumin. J. Sep. Sci..

[60] Kalbac M., Hsieh Y.P., Farhat H., Kavan L., Hofmann M., Kong J., Dresselhaus M.S. (2010). Defects in Individual Semiconducting Single Wall Carbon Nanotubes: Raman Spectroscopic and in Situ Raman Spectroelectrochemical Study. Nano Lett..

[61] Dresselhaus M.S., Dresselhaus G., Saito R., Jorio A. (2005). Raman Spectroscopy of Carbon Nanotubes. Phys. Rep..

[62] Ramos S.C., Vasconcelos G., Antunes E.F., Lobo A.O., Trava-Airoldi V.J., Corat E.J. (2010). Wettability Control on Vertically-Aligned Multi-Walled Carbon Nanotube Surfaces with Oxygen Pulsed DC Plasma and CO_2_ Laser Treatments. Diam. Relat. Mater..

